# Current Perspectives on High-Throughput Sequencing (HTS) for Adventitious Virus Detection: Upstream Sample Processing and Library Preparation

**DOI:** 10.3390/v10100566

**Published:** 2018-10-16

**Authors:** Siemon H. Ng, Cassandra Braxton, Marc Eloit, Szi Fei Feng, Romain Fragnoud, Laurent Mallet, Edward T. Mee, Sarmitha Sathiamoorthy, Olivier Vandeputte, Arifa S. Khan

**Affiliations:** 1 Product Research and Development, Analytical Sciences, Sanofi Pasteur, Toronto, ON L4J 7Z4, Canada; sarmitha.sathiamoorthy@gmail.com; 2 Biogen, Inc., Research Triangle Park, NC 27709, USA; cassandra.braxton@biogen.com; 3 PathoQuest, 75015 Paris, France; marc.eloit@pathoquest.com; 4 Institute Pasteur, Biology of Infection Unit, Inserm U1117, Pathogen Discovery Laboratory, 75015 Paris, France; 5 Merck & Co., Inc., West Point, PA 19486, USA; szifei_feng@merck.com; 6 Texcell, Genavenir 5 1, Rue Pierre Fontaine, 91058 évry, France; rfragnoud@texcell.fr; 7 Product Research and Development, Analytical Sciences, Sanofi Pasteur, 69280 Marcy L’Etoile, France; laurent.mallet@sanofi.com; 8 Division of Virology, National Institute for Biological Standards and Control, Medicines and Healthcare Products Regulatory Agency, South Mimms, Hertfordshire EN6 3QG, UK; edward.mee@nibsc.org; 9 Analytical Research and Development, GSK, 1330 Rixensart, Belgium; olivier.x.vandeputte@gsk.com; 10 Division of Viral Products, Office of Vaccines Research and Review, Center for Biologics Evaluation and Research, U.S. Food and Drug Administration, Silver Spring, MD 20993, USA; arifa.khan@fda.hhs.gov

**Keywords:** adventitious virus, virus detection, high-throughput sequencing, next-generation sequencing, sample preparation, biologics

## Abstract

A key step for broad viral detection using high-throughput sequencing (HTS) is optimizing the sample preparation strategy for extracting viral-specific nucleic acids since viral genomes are diverse: They can be single-stranded or double-stranded RNA or DNA, and can vary from a few thousand bases to over millions of bases, which might introduce biases during nucleic acid extraction. In addition, viral particles can be enveloped or non-enveloped with variable resistance to pre-treatment, which may influence their susceptibility to extraction procedures. Since the identity of the potential adventitious agents is unknown prior to their detection, efficient sample preparation should be unbiased toward all different viral types in order to maximize the probability of detecting any potential adventitious viruses using HTS. Furthermore, the quality assessment of each step for sample processing is also a critical but challenging aspect. This paper presents our current perspectives for optimizing upstream sample processing and library preparation as part of the discussion in the Advanced Virus Detection Technologies Interest group (AVDTIG). The topics include: Use of nuclease treatment to enrich for encapsidated nucleic acids, techniques for amplifying low amounts of virus nucleic acids, selection of different extraction methods, relevant controls, the use of spike recovery experiments, and quality control measures during library preparation.

## 1. Introduction

A variety of high-throughput sequencing (HTS) platforms (also known as massively parallel sequencing (MPS) or next generation sequencing (NGS) have been developed (e.g., Roche 454 (discontinued), Illumina, Ion Torrent, PacBio, Oxford Nanopore) that can generate millions of nucleic acid bases within a single sequencing reaction. The large amount of data from a single sample gives HTS platforms the potential to detect viral nucleic acid that may be present at a low level in a complex biological sample. Investigations of various sample types including clinical, environmental, and biological, have demonstrated the capabilities of HTS for broad virus detection and novel virus discovery.

The capabilities of HTS for the broad detection of known and novel viruses suggests its potential value in assuring the absence of unintended microbial agents such as adventitious viruses, which is important for ensuring product safety and quality. Such agents could potentially be introduced at different steps in the manufacturing process from various sources, for example, due to previously undetected agents in cell banks or viral seeds, viruses associated with source species for raw materials, or from the environment [1]. Regulatory authorities, such as the US Food and Drug Administration (FDA), European Medicines Agency (EMA), and the World Health Organization (WHO), publish guidance documents and national pharmacopeias publish monographs that include recommendations or requirements for the testing of adventitious agents for the safety of biological products [2,3,4]. Although the currently recommended adventitious agent tests are extensive and generally used at multiple stages of manufacturing, they have limitations due to the biological properties of some viral families so they may not be detected by the currently recommended in vivo or cell culture assays, or due to genetic viral diversity so that they are only partly or not detectable by PCR assays: this incomplete coverage may allow some adventitious viruses to go undetected during the manufacturing process. For example, research investigations of some vaccines using HTS and virus microarrays revealed the presence of porcine circovirus type 1 (PCV1) nucleic acid in a licensed rotavirus vaccine [5], despite previously having passed all compendial tests during development, clinical trials, and production [6]. Fortunately, the presence of PCV1 was not considered a threat to human health [7]. More recently, HTS and degenerate PCR assays revealed the presence of a novel rhabovirus in the Sf9 parent cell line, which is used for the development of various baculovirus-expressed products; however, it was shown that the virus did not replicate in human cells [8]. Such virus discoveries exemplify the potential of HTS to enhance the safety of biologics by complementing the current adventitious agent testing strategies for some biological samples, for example, cell banks, viral seeds, or raw materials, with the possibility of replacing some of the tests in the future. This topic was the focus of a recent international conference on “Next generation sequencing for adventitious virus detection in biologics cosponsored by the International Alliance for Biological Standardization (IABS) and FDA [9].

**Figure 1:**
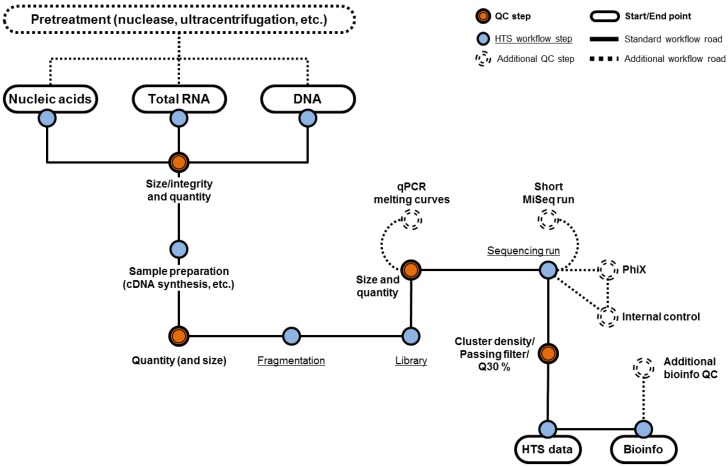
Different approaches for high-throughput sequencing (HTS) sample preparation and potential quality control (QC) opportunities. Experimental steps and potential QC steps are represented by blue and orange circles respectively. The minimum steps for the detection of adventitious viruses are connected by solid lines with optional QC procedures connected by dashes. Figure 1 starts with potential pre-treatment of the sample prior to nucleic acid extraction (e.g., total nucleic acid, total RNA or total DNA extractions). The extracted material is assessed for size, integrity, and quality to ensure sufficient amount of input material. After sample preparation the integrity of DNA and the quality of the library is again assessed. At this point, the quality of the sequencing library can be confirmed by doing qPCR or a small scale sequencing run (e.g., MiSeq) prior to a full-scale sequencing run to save cost and time. Additionally, internal controls can be included into the sample as a way to monitor the performance of the sample preparation and sequencing.

One essential component of an effective HTS assay for the evaluation of biological products is the sample preparation strategy that needs to be broad and efficient for the extraction of nucleic acid from viruses with different physical properties that influence their sensitivity or resistance to various chemical treatments. Viruses can be enveloped or non-enveloped, with varying virion size and morphology. Viral genomes can be single-stranded or double-stranded DNA or RNA, range from a few thousand bases to millions of bases, and DNA genomes can be linear or circular. Therefore, an important control is to evaluate the efficiency of the nucleic acid extraction method by using model viruses representing different physicochemical properties. Recent viral metagenomics studies have focused on RNA virus samples of specific origin (e.g., human skin or stool samples) or ecosystems (e.g., water or soil samples) [10,11,12,13,14]. Depending on the starting matrix and the intended application (e.g., transcriptome versus virome sequencing), sample preparation might include different techniques such as nuclease treatment to enrich for particle-associated viral sequences, steps for amplification of low amounts of viral nucleic acids, and the selection of best-suited extraction technologies. In addition, different starting matrices may require different modifications to the sample preparation pipeline to ensure maximum extraction efficiency of viruses and should include assay controls and spike recovery experiments. An overview of the different potential upstream processing steps and quality control checkpoints in an HTS workflow is shown in Figure 1.

This paper presents our perspectives on upstream sample processing and library preparation for the broad detection of adventitious viruses by HTS based on discussions in the Advanced Virus Detection Technology Interest Group (AVDTIG) [15].

## 2. Comparison of Extraction Methods for Viral Nucleic Acid Detection

Efficient extraction of nucleic acids from viruses is a critical sample preparation step for assessing adventitious viral agents using HTS and can be impacted by different physical properties such as viral genome type, genome size, and the presence or absence of viral envelopes. An ineffective extraction procedure may limit HTS detection of specific types of viruses, thus leaving gaps in the adventitious agent testing strategy. Ideally, both RNA and DNA (single-strand and double-strand, linear and circular, small size, and large size) should be extracted efficiently to ensure the broadest detection and highest data yield.

Different nucleic acid extraction methods have been shown to vary in performance [16,17,18]. Eric Delwart’s group compared silica membrane-based, bead-based, and phenol:chloroform-based extraction methods and found that phenol:chloroform gave the lowest extraction efficiency, while silica columns yielded the best results [17]. An independent study by Sanofi Pasteur (Toronto, ON, Canada) using different extraction kits also showed silica membrane-based methods as the most efficient for extracting single-stranded and double-stranded RNA and DNA from viral particles [18]. It was also noted that organic extraction of viral nucleic acids yielded the worst recovery of double-stranded viral DNA. However, the double-stranded RNA virus was highly recovered in comparison to the other extraction methods. In contrast, a study from Merck & Co. (West Point, PA, USA) demonstrated improved recovery using phenol-chloroform extraction when compared to a silica membrane-based extraction kit [19].

The plenitude and breadth of nucleic acid recovery, a very important first step to sample preparation, will influence the overall sensitivity of virus detection by an assay. The selection of the extraction methods can best be evaluated in the context of the entire pipeline using a panel of different virus types to determine its sensitivity before the implementation of any specific pipeline [20,21].

## 3. Nuclease Treatment for the Isolation of Particle-Associated Viral Nucleic Acids

Use of a nuclease step prior to nucleic acid extraction can be useful to enrich for viral particles (e.g., encapsidated viral genomes). Nuclease treatment helps to reduce background sequences of non-encapsidated nucleic acids (e.g., host cell DNA/RNA, “free” viral genomes) that may be present in samples such as cell lysates, culture supernatants, viral seeds, and raw materials. The reduction of host cell nucleic acids increases the sensitivity of viral detection, which is mainly based on the ratio of virus/host sequences, and reduces the complexity of data analysis [21]. This strategy is also beneficial for potentially distinguishing the presence of encapsidated (and possibly infectious) viral particles against the non-infectious, naked, nucleic acids. It is conceivable that nuclease treatment conditions (which may include pH, salt, heat, and inactivation conditions) may have adverse effects on the viral genome during the nuclease treatment (in particular, if the viral capsid is already damaged). However, reduction in one or more specific viral categories may not preclude use of nuclease treatment, especially if there is also a decrease in the background signal which may result in an increase in the overall sensitivity for the matrix. Therefore, it is prudent to conduct spiking studies using several structurally-distinct viruses to assess the effects of nuclease treatment condition on viral genome recovery [21]. Spike recovery experiments are discussed in more detail below. When incorporating nuclease treatment into the sample-processing workflow, spike recovery experiments should be based on nuclease-treated genome copy numbers of the viral stocks to accurately assess recovery. For viral stocks that are not highly purified, there may be free nucleic acids present that can affect quantitation of recovery. Therefore, it is important to determine the total genome copy number and nuclease-resistant genome copy number for each viral stock by characterizing it without and with nuclease treatment, respectively. Inactivation of nucleases before a lysis step is critical in order to minimize any potential nucleic acid degradation of subsequently lysed viral particles.

One possible control to monitor the effects of nuclease treatment on reducing or increasing potential viral contaminant detection is to include a control sample spiked with a low-level of a known virus prior to digestion and extraction. Another readout could be infectivity assays, that indirectly assess the impact on genomes associated with infectious virus particles. The choice of spike virus should be based on the pre-determined knowledge of its sensitivity to the nuclease or on a risk assessment to identify those viruses most likely to be encountered in that particular sample. Reduction in one or more specific viral categories may not preclude use of nuclease treatment, especially if there is also a decrease in the background signal which may result in an increase in the overall sensitivity for the matrix. The impact of the nuclease treatment on different virus families may need to be considered by risk assessment since the reduction in the background sequences can results in an overall increase in sensitivity of virus detection by HTS.

## 4. Whole Genome Amplification

Whole genome amplification (WGA) is a technique used to increase the amount of nucleic acids extracted from a sample. It has potential application for nucleic acid preparation when the starting biological input sample contains a limited amount of genetic material (e.g., few cells or very few viral particles) and yields insufficient quantities of nucleic acid for library preparation. Currently, there are multiple techniques for whole genome amplification such as degenerate oligonucleotide primed PCR (DOP-PCR) [22] or an isothermal amplification using a randomly fragmented genome, Multiple Annealing and Looping-Based Amplification Cycles (MALBAC) [23] and multiple strand displacement amplification (MDA) [24,25]; for example using Phi29 DNA polymerase that can synthesize upward of 100,000 bases without falling off the template DNA and also possesses a very low error rate compared to that of Taq polymerase [24]. DOP-PCR gave a large number of viral reads but was strongly biased so that prominent viruses were not being detected after using DOP-PCR [17]. The detection of adventitious viruses using HTS can be influenced by the sample type; for example, sample extraction can yield very low amounts of nucleic acids from some starting materials such as media, FBS and other raw materials. In these situations, WGA can be a suitable technique to amplify the extracted nucleic acid to yield sufficient material for the creation of an HTS library.

A comparison of sequencing data that was generated with and without MDA, where MDA was carried out with random hexamers, to synthesize DNA using the DNA polymerase, Phi29 was conducted by Sanofi Pasteur [18]. In addition to facilitating the creation of a sequencing library, in some cases, the detection sensitivity as determined by HTS towards some viruses was higher when WGA was used during sample preparation when the amount of nucleic was very low. For these viruses, the higher number of reads also increases the genome coverage of the detected viral signal, suggesting more sequence complexity in the sequencing library. While WGA can be beneficial in some cases, some potential biases against RNA viruses were observed when using MDA. This bias is likely due to the inefficiencies of the reverse transcription step (which would be similar to those for qPCR) and Phi29’s preference towards longer DNA molecules as its template [18].

One potential technique to overcome the bias against short nucleic acid fragments could be the ligation of cDNA fragments into longer DNA molecules prior to amplification by Phi29 [26]; however, performing this ligation will produce chimera sequences that need to be accounted for in the subsequent bioinformatics analysis of the sequencing results. In addition, it is important to note that there is a potential for biased amplifications of DNA across the genome that potentially needs to be accounted for in the data analysis.

Another approach might be considered for samples that have very low total nucleic acid levels. Often, libraries made from such samples do not meet conventional quality control (QC) specifications (see quality control section below). The suitability of a low-nucleic-acid library could be evaluated the same way one would evaluate a PCR test that has a very wide dynamic range—that is by evaluating recovery of a dilution series of spike viruses. If the recovery is sufficient (e.g., adequate recovery of the spiked viruses), despite low input nucleic acid quantities, then the library could be considered valid and entirely useful even if it does not meet all QC specifications. Nevertheless, library preparation needs proper validation for use within a GMP environment.

## 5. Quality Control for Upstream Steps in HTS

The quality of the input material used for constructing the HTS library will impact the quality of the sequencing run and potentially the subsequent detection of the viral nucleic acids. For instance, if the library fragments are short, the sequencing will terminate prematurely, generating shorter sequencing reads that result in less data (information) being produced. For some sequencing platforms, shorter fragments also cluster more efficiently and generate an additional bias for shorter reads. If library concentration is over or under estimated, suboptimal clustering will occur, resulting in a reduction in the volume of data generated. If limited amounts of starting material are available and if no amplification of the starting material (e.g., by whole genome amplification) is performed, QC will likely be limited to the QC of only the library, which will indirectly reflect the quality of the starting material. A consequence of using limited amounts of starting material is that HTS steps will not be performed under optimal conditions, and there may be a higher risk of a sequencing run generating suboptimal data size not representative of the full complexity of the sample.

### 5.1. QC for Assessing RNA

Assessing the quality of the starting RNA material (e.g., for transcriptome) that will be used in subsequent HTS steps is important as it will influence the data output. Ideally, total RNA should be checked for integrity and purity using a microfluidics electrophoresis assay (e.g., Bioanalyzer (Agilent, Santa Clara, CA, USA), TapeStation (Agilent, Santa Clara, CA, USA) or LabChip GX (PerkinElmer, Waltham, MA, USA)) and evaluating the RNA integrity number or by examining the absorbance ratios. Recent developments in the deconvolution of absorbance spectra of nucleic acids contaminated with different reagents used for the purification of nucleic acids, help in identifying contaminants, and obtaining corrected concentration results. Contaminants are critical negative factors in RNA samples since they may inhibit subsequent enzymatic steps converting RNA to double stranded cDNA, reducing cDNA yield and quality [27]. Checking for inhibition can be performed by using a known internal control, such as the External RNA Controls Consortium (ERCC) controls developed under the National Institute of Standards and Technology (NIST) supervision [28,29].

### 5.2. QC for Assessing DNA

The same principles for high quality RNA as starting materials also hold true for DNA samples, although 260 nm/280 nm ratios are generally closer to 1.8. Integrity of DNA can be checked by agarose gel electrophoresis (when available in sufficient quantity) or by using a microfluidic electrophoresis system (e.g., Bioanalyzer). No smearing of the DNA sample is expected, as DNA degradation during extraction is less frequent than with RNA. The pH of the solution in which the nucleic acids are eluted, diluted, and stored has an impact on the absorbance of the sample. This may result in an under (acidic conditions) or an over (basic conditions) estimation of the ratios therefore the use of buffered elution solutions is highly recommended. When using a spectrophotometer to assess the DNA quality, it is important to understand that the wavelength accuracy of the spectrophotometer can have a large effect on the optical densities and consequently on the 260/280 ratio. Additionally, the sample may need to be diluted or concentrated to obtain a range for accurate measurement.

### 5.3. Assessing the Starting Material for the Sequencing Library

Nucleic acids should be quantified to introduce the appropriate amount of material into the library preparation workflow. Very often nucleic acids are not quantifiable after nuclease treatment or are well below the amounts required for the preparation of libraries. Nucleic acid quantification can be achieved either by UV spectrophotometry or fluorescence measurement. Considering the lack of specificity of UV methods, fluorescence methods based on the use of nucleic acid biding/intercalating dyes, which are by far more specific and sensitive, are favored. Nucleic acids are then directly fragmented (DNA or sometimes RNA) or can go through additional preparation steps (ribosomal RNA depletion, poly A+ selection, specific enrichment, conversion of RNA into cDNA, whole genome or transcriptome amplification, etc.) before fragmentation. These additional steps are commonly checked for quality by using the recommendations (if any) of the kit or reagent providers or through the use of internal controls. Additional controls should include assays to determine the size of the nucleic acids and their quantity using the methods and cautions already mentioned. Fragmentation of DNA or cDNA can be performed either mechanically (ultrasonication), enzymatically (e.g., using Fragmentase or via transposase/transposon complexes) or chemically (heat and magnesium for RNA) [30]. The fragmentation of nucleic acids also comprises additional purification steps aimed at discarding fragments, which are too long or too short with solid-phase reversible immobilization (SPRI) beads. Checking that the fragments are of the expected size is of paramount importance for the success of the sequencing run. This can be achieved by visualizing the size profile of the fragmented nucleic acids using a microfluidic electrophoresis system (e.g., Bioanalyzer) and purifying the sample if necessary. Classical electrophoresis is still used because it allows simultaneous visualization and extraction of the region of interest but leads to a risk of cross contaminations between samples; it has also been demonstrated that subsequent purification of the fragmented material from the gel reduces the representation of AT-rich regions [30].

### 5.4. Assessing Sequencing Library Preparation

Library construction is a multi-step process which can rely on end-repair, A-tailing, adaptor ligation and limited PCR amplification and end with a QC step consisting of determining the size of the DNA fragments and their quantity. The fragment size distribution and quantity of the sequencing library can be checked by using a microfluidics electrophoresis assay (e.g., Bioanalyzer, TapeStation or LabChip GX), while quantity can also be evaluated by qPCR (using kits specifically designed to amplify DNA fragments flanked by the sequencing adaptors) or by fluorescence assays (e.g., Qubit (ThermoFisher, Waltham, MA, USA) or Picogreen (ThermoFisher, Waltham, MA, USA)). Quantification assays based on digital droplet PCR have been evaluated and shown to be valuable complements to fluorescence assays (Qubit, Picogreen and qPCR) [31,32].

When performing library quantification by qPCR, it is advisable to include an internal control such as a previously sequenced library, a PhiX control library prepared by a provider or a commercially available control that is processed the same way as the prepared library. Samples should be processed in replicates and tested at different dilutions to obtain raw quantification data that fall into the dynamic range of the assay. An interesting additional control may be to analyze the melting curves of the qPCR amplicons in order to assess adapter-dimer carry-over in the libraries as these adapter-dimers can interfere with clustering on the Illumina platform.

### 5.5. Negative Controls

Appropriate no-template controls should be considered for different stages of sample preparation. Inclusion of a no-template control could give information regarding irrelevant signals from reagents. Silica membranes have been found to contain contaminating viral nucleic acids [33,34] and recombinant enzymes used during the sample preparation steps can also contain host nucleic acid.

### 5.6. Conclusion for QC

Ultimately, the assessment of the starting material used in each step of a HTS workflow (i) has to show that the quality and integrity of the nucleic acids are compatible with the intended use and (ii) provide some indication as to the quality of the HTS outcome. It should also be noted that a failed QC is not necessarily indicative that the HTS run will fail but is clearly associated with low quality HTS outcomes. This does not mean that the HTS data will not be of value; instead, additional bioinformatics clean-up and analysis will be required to bring out the exploitable data. Before using their libraries in HTS runs, some labs perform a short MiSeq (or equivalent) run as a final QC step to evaluate the quality of their HTS workflows and the effectiveness of their QC steps.

## 6. Evaluating the Performance of High-Throughput Sequencing for Adventitious Virus Detection

Spike recovery experiments using model viruses representing the biophysical diversity of known viral families are key for assessing the performance of high-throughput sequencing for detection of adventitious viruses. The objective of these studies is to carry out the entire sample preparation pipeline and assess different conditions to determine whether there are conditions that would lead to a biased recovery of viruses in the spike panel and to understand this impact across the different viruses. If there is evidence of reduction in recovery, then specific controls might need to be incorporated in the study in order to further evaluate the cause for the reduction in recovery.

For a quantitative assessment of the recovery, viral stocks used for spike recovery experiments should ideally be highly purified and characterized for genome content. It is important to use stocks that are relevant to cases of viral contaminations. In particular, viral stocks grown in cell culture and immediately frozen may contain high amounts of non-encapsidated nucleic acids.

At present five viral stocks have been prepared and characterized by the FDA through ATCC (American Type Culture Collection, Manassas, VA 20110, USA) and are available as a quantitative reference material for sequence-based adventitious virus detection. In addition, a multiplexed viral pool from NIBSC can be used as a qualitative material [20]. Furthermore, the need for other types of virus reference materials is under discussion in the AVDTIG.

In addition, evaluation of the performance of NGS for detecting infected cell lines can be carried out by using a dilution of infected and non-infected cells. Infected cells can have a productive infection (i.e., those which produce mature virus particles associated with cells and/or released into the supernatant) or a latent infection by viruses that are integrated and expressing some transcripts or viral genomes present as an episome that may potentially produce viruses at a later stage. Spiking viral particles into cell lysates is not a representative test sample for infected cells and could deeply underestimate the sensitivity of HTS. Transcriptomic analysis by NGS is one potential approach for the detection of viral RNA that are transcribed during productive or latent cycles. Such studies are currently under discussion in the AVDTIG [35].

## 7. Other Considerations

It is important to note that qPCR is not impacted by the number of background host nucleic sequences in the same way as HTS. In addition, PCR or qPCR methods are often validated in “best case” conditions where primers perfectly match the strain used for spiking. One key advantage of HTS when comparing between HTS and PCR/qPCR is the potential to detect distantly related or unknown viruses. While this may be accomplished by PCR/qPCR by using non-specific rather than specific primers, detection is not guaranteed when the sequence divergence is great.

Although out-of-scope for this discussion, the choice of sequencing platform and instrument, reference database, and data analysis pipeline will impact the performance and sensitivity of using HTS for adventitious virus detection. Selecting an instrument with higher throughput might allow for more tolerance for background signals. A comprehensive reference database is also critical for the accurate determination of the adventitious virus signal [36]. Additionally, a database with well-annotated sequences will facilitate obtaining accurate and interpretable results, whereas, analysis using a larger reference database containing irrelevant sequences will require more computational power and storage Finally, the design of the bioinformatics analysis is crucial for accurate detection and identification of any viral contaminants and are under discussion in the AVDTIG [35].
